# Identification of S-nitrosylation sites based on multiple features combination

**DOI:** 10.1038/s41598-019-39743-9

**Published:** 2019-02-28

**Authors:** Taoying Li, Runyu Song, Qian Yin, Mingyue Gao, Yan Chen

**Affiliations:** Department of Maritime Economics and Management, Dalian Maritime University, No. 1 Linghai Road, Dalian, 116026 China

## Abstract

Protein S-nitrosylation (SNO) is a typical reversible, redox-dependent and post-translational modification that involves covalent modification of cysteine residues with nitric oxide (NO) for the thiol group. Numerous experiments have shown that SNO plays a major role in cell function and pathophysiology. In order to rapidly analysis the big sets of data, the computing methods for identifying the SNO sites are being considered as necessary auxiliary tools. In this study, multiple features including Parallel correlation pseudo amino acid composition (PC-PseAAC), Basic kmer1 (kmer1), Basic kmer2 (kmer2), General parallel correlation pseudo amino acid composition (PC-PseAAC_G), Adapted Normal distribution Bi-Profile Bayes (ANBPB), Double Bi-Profile Bayes (DBPB), Bi-Profile Bayes (BPB), Incorporating Amino Acid Pairwise (IAAPair) and Position-specific Tri-Amino Acid Propensity(PSTAAP) were employed to extract the sequence information. To remove information redundancy, information gain (IG) was applied to evaluate the importance of amino acids, which is the information entropy of class after subtracting the conditional entropy for the given amino acid. The prediction performance of the SNO sites was found to be best by using the cross-validation and independent tests. In addition, we also calculated four commonly used performance measurements, i.e. Sensitivity (Sn), Specificity (Sp), Accuracy (Acc), and the Matthew’s Correlation Coefficient (MCC). For the training dataset, the overall Acc was 83.11%, the MCC was 0.6617. For an independent test dataset, Acc was 73.17%, and MCC was 0.3788. The results indicate that our method is likely to complement the existing prediction methods and is a useful tool for effective identification of the SNO sites.

## Introduction

Protein post-translational modifications play a very important role in the processing of protein, protein maturation, as well as altering the physical and chemical properties of proteins. As a result, the space conformation, three-dimensional location and the stability of the proteins are likely change, which can lead to the function alteration. Moreover, the structural features of the modified groups can produce a far-reaching impact on the properties, as well as, the functions of proteins. In 1998^1^, the Nobel Prize for Physiology or Medicine was rewarded for breakthrough discoveries that showed nitric oxide to be a freely-diffusible signaling molecule and a secondary messenger. NO plays a vital role in the cardiovascular system^2^. It is noticed that S-nitrosylation (SNO) is the covalent interaction of nitric oxide with the thiol group of cysteine residues^1,3^ and is well characterized as a major source of NO bioactivity^4^. Many experimental methods have been applied for distinguishing the SNO sites, such as the biotin-switch technique (BST)^5,6^, SNO-Cys site identification (SNOSID)^7–9^, and the resin-associated capture (RAC)^10^. These experimental methods have successfully provided a very effective information in identifying the SNO sites. The BST was designed to purify and detect the SNO proteins, mainly composed of three principal steps: (i) The methylthiolation of free cysteine thiols with methyl methanethiosulfonate (MMTS); (ii) Reduction of SNOs to thiols with ascorbate; (iii) Ligation of the nascent thiols with N-[6-(Biotinamido)hexyl]-3′-(2′-Pyridyldithio)-propionamide (biotin-HPDP)^11^. In combination with the traditional mass spectrometry (MS), BST has indeed contributed to discovering a lot of potential protein SNO sites^12–15^. A proteomic method called SNOSID, that identified the endogenous and chemically-induced SNOs in the proteins from tissues or cells, was also developed to determine the potential SNO sites on the cysteine residues in complex protein mixtures. Furthermore, RAC based method was also developed to detect the SNO proteins^10^. In 2009, Foster *et al*.^16^, explored a protein microarray-based approach to screen the SNO sites. These methods made great contributions to the development of the prediction of SNOs, however, to a certain degree, they were considered to be time-consuming and also had a relatively low throughput data. Recently, several machine learning approaches have been proposed and have provided helpful information being used for further experimental verification of the protein SNO sites. Hao *et al*.^7^ developed a prediction tool for the SNO sites, which was based on the support vector machine (SVM)^17^ algorithm, and used a training dataset that consisted of 65 positive SNO sites and 65 non-SNO sites. A few years later, Xue *et al*.^2^ proposed a method called GRS-SNO by using a group-based predicting system based on 504 experimentally verified SNO sites in 327 unique proteins. Shortly afterward, Li *et al*.^18^ established the predictor CPR-SNO and built a web server based on a coupling pattern encoding scheme. Xu *et al*.^19,20^ developed the iSON-AApair that takes into account, the effects of sequence correlation. More recently, Jia *et al*.^21^ used an Adapted Normal Distribution Bi-Profile Bayes (ANBPB) and Chou’s PseAAC composition constituting the feature vector. The composition of Zhang *et al*.^22^ were also based on the Chou’s PseAAC, by incorporating the various sequences derived feature.

Each of the above mentioned methods had their own advantage, as well as, played an important role in the research for prediction of protein S-nitrosylation sites. However, it is noted that the prediction performance is not really satisfactory. Therefore, there is necessity to discover more efficient methods for the SNO sites identification.

In this study, we extracted nine types of features, including PC-PseAAC (25), kmer1 (20), kmer2 (400), PC-PseAAC_G (25), ANBPB (40), DBPB (38), BPB (40), IAAPair (39) and PSTAAP (18). In order to remove the redundant information, the information gain (IG) method was applied to select the features. Finally, the optimization of 425D feature vector (PC-PseAAC (25), kmer1 (20), kmer2 (180), PC-PseAAC_G (25), ANBPB (40), DBPB (38), BPB (40), IAAPair (39), and PSTAAP (18) was used to construct our prediction model. Our results suggest that IG can provide an improved performance, which is comparable to the one without the use of the IG method. The results indicated that selecting the IG feature is a promising method to predict the features with high dimension with the SNO sites.

## Results and Discussion

### Combination of different features

To evaluate the performances of the combined feature sets for sorting SNO sites and non-SNO sites, we tested the prediction performances on the Jackknife test^23^, which is considered as the most objective and always yields a unique result for a given dataset^21,24^. The combined features were composed of the PC-PseAAC, kmer1, kmer2, PC-PseAAC_G^25^, ANBPB^21^, DBPB, BPB^26^, IAAPair^19^, and PSTAAP^20,27^ models and the detailed results are shown in Supplementary Table S1. The results show that the prediction performance was enhanced through the combined features. As shown in Table 1, PC-PseAAC with the Acc of 62.82% was regarded as the basic features, and was then incorporated to kmer1 to improve the prediction performance, which reached the Acc of 64.83%. Secondly, combination of features PC-PseAAC + kmer1 were further incorporated with the component of kmer2 one by one, and new combined features PC-PseAAC, kmer1 and kmer2 reached Acc of 64.89%. This process was terminated at feature combination PC-PseAAC, kmer1, kmer2, PC-PseAAC_G, ANBPB, DBPB, BPB, IAAPair, and PSTAAP, which increased the Acc to 74.24% and MCC^17,28–30^ to 0.4837. From the above, it can be concluded that the combined features can improve Acc of 11.42%. The parameters λ and the weight factor w were found to offer the best results for the features PC-PseAAC and PC-PseAAC_G and the optimized values were λ = 5 and w = 0.5.

**Table 1:** Performance of the combination feature with different sequence encoding schemes in jackknife test.

Dimension	Sequence encoding schemes	Sn (%)	Sp (%)	Acc (%)	MCC
25	PC-PseAAC	72.33	54.32	62.82	0.2699
45	PC-PseAAC + kmer1	68.67	61.36	64.83	0.3004
445	PC-PseAAC + kmer1 + kmer2	64.3	65.43	64.89	0.297
470	PC-PseAAC + kmer1 + kmer2 + PC-PseAAC_G	64.57	65.43	65.02	0.2997
510	PC-PseAAC + kmer1 + kmer2 + PC-PseAAC_G + ANBPB	65.53	72.84	69.37	0.3848
548	PC-PseAAC + kmer1 + kmer2 + PC-PseAAC_G + ANBPB + DBPB	65.94	72.84	69.57	0.3888
588	PC-PseAAC + kmer1 + kmer2 + PC-PseAAC_G + ANBPB + DBPB + BPB	65.94	72.96	69.63	0.3901
607	PC-PseAAC + kmer1 + kmer2 + PC-PseAAC_G + ANBPB + DBPB + BPB + IAAPair	66.48	73.21	70.02	0.3979
645	PC-PseAAC + kmer1 + kmer2 + PC-PseAAC_G + ANBPB + DBPB + BPB + IAAPair + PSTAAP	73.46	74.94	74.24	0.4837`

### Features selection via IG

**Figure 1:**
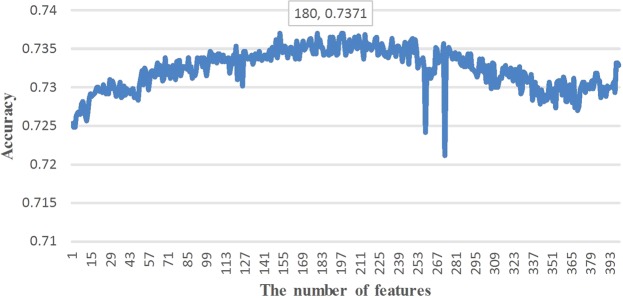
The predictive performance of different models based on incremental feature selection of features sorted by IG.

**Figure 2:**
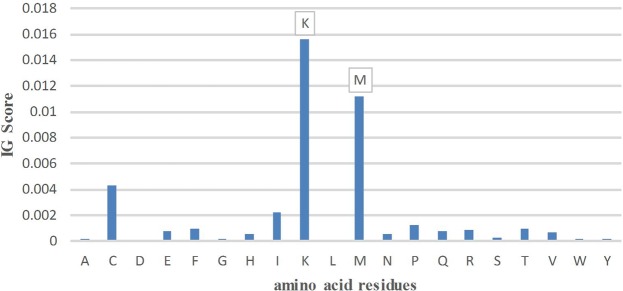
The IG score of each amino acid residues.

**Figure 3:**
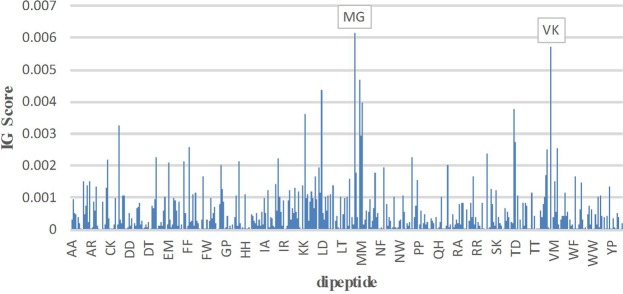
The IG score of each dipeptide.

To further improve the prediction performance, these features were optimized based on the above-mentioned IG optimization method. The four types of features PC-PseAAC, kmer1, kmer2, and PC-PseAAC_G are mainly related to the frequency of amino acids but are independent of the position of protein sequences. Hence, we optimized these four types of features based on the IG score of the amino acid residues. Firstly, we sorted the importance of amino acid composition (AAC) and the amino acid pair composition (i.e. kmer2) by IG score, and then applied the incremental feature selection to find out the best feature subset for maximizing prediction performance. According to the final performance evaluation, the application of IG score on kmer2 was especially distinguishable. The detailed prediction performances for different number of features combination on 10-fold cross-validation were shown in Fig. 1. It can be seen that when the dimension for the feature vector selected to be 180, the predictive performance achieved the highest value with Sn of 72.79%, Sp of 74.64%, Acc of 73.71%, and MCC of 0.4741. However, there was no obvious improvement for the other three types features PC-PseAAC, kmer1, and PC-PseAAC_G. This could be due to the low dimensions of these three types of features (less than 50). On the contrary, the dimension of kmer2 was 400, and the feature matrix was an extremely sparse matrix and hence having IG reflecting a good performance. The results of the IG score ranking importance of amino acid residues and dipeptide are displayed in Fig. 2 and Fig. 3, respectively and the detailed results are shown in Supplementary Table S2. It is noteworthy that the amino acid residues K, M, and C and the dipeptides MG, VK, and ML exhibited a great contribution to the prediction performance. Fig. 2 and Fig. 3 showed that the highest IG score reached 0.0156, 0.0112 and 0.0043 for the amino acid residues K, M, and C, respectively, while the highest IG score reached 0.0062, 0.0057 and 0.0047 for the amino acid dipeptides MG, VK, and ML, respectively.

Before the features selection, the prediction performance with the Sn of 73.46%, the Sp of 74.94%, and the Acc of 74.28%. After removing the irrelevant feature and then determining the optimal combination of features, we then obtained the best prediction performance with the Sn of 73.60%, the Sp of 75.93% and the Acc of 74.82, respectively. As can be seen, all of the three measurements have been improved slightly. But the prediction performance was not satisfied, it need us to make improvements on this work in the future. The results for the best predictive performance are shown in Table 2. An improved predictive Acc for the models that were trained with the optimized features was being seen when compared with the model with non-optimized features. As given in Supplementary Table S2, such 425 [PC-PseAAC(25) + kmer1(20) + kmer2(180) + PC-PseAAC_G(25) + ANBPB(40) + DBPB(38) + BPB(40) + IAAPair(39) + PSTAAP(18)] features regarded as the optimal feature set for the selected model. Based on the 425 features, the predictive Sn, Sp, and Acc were 73.60%, 75.93% and 74.82%, respectively. These results indicate that the key amino acid residues and the key dipeptide used in optimizing the models can enhance the prediction performance of the SNO sites. Consequently, the features combined with key amino acid residues were applied to implement a novel and high-performance tool for identifying cysteine S-nitrosylated sites.

**Table 2:** Features optimization based on IG on Jackknife test.

IG Dimension	Sequence encoding schemes	Sn (%)	Sp (%)	Acc (%)	MCC
645	PC-PseAAC + kmer1 + kmer2 + PC-PseAAC_G + ANBPB + DBPB + BPB + IAAPair + PSTAAP	73.46	74.94	74.24	0.4837
425	PC-PseAAC + kmer1 + **kmer2** + PC-PseAAC_G + ANBPB + DBPB + BPB + IAAPair + PSTAAP	73.60	75.93	74.82	0.4952
425	**PC-PseAAC** + kmer1 + kmer2 + PC-PseAAC_G + ANBPB + DBPB + BPB + IAAPair + PSTAAP	73.60	75.93	74.82	0.4952
425	PC-PseAAC + **kmer1** + kmer2 + PC-PseAAC_G + ANBPB + DBPB + BPB + IAAPair + PSTAAP	73.60	75.93	74.82	0.4952
425	PC-PseAAC + kmer1 + kmer2 + **PC-PseAAC_G** + ANBPB + DBPB + BPB + IAAPair + PSTAAP	73.60	75.93	74.82	0.4952

Bold blackbody is the feature extracted by IG method.

### Comparison with other feature selection methods

In this paper, different feature selection methods were exploited for comparison. We made several comparisons for evaluating the performance of IG with Max-Relevance-Max-Distance^31^ (MRMD), a method for feature selection. MRMD contains two components, max distance and maximal relevance. The max distance selects a new feature which has the least redundancy in the residual of features, while the maximal relevance selects feature that has the strongest relevance to the target class.

**Figure 4:**
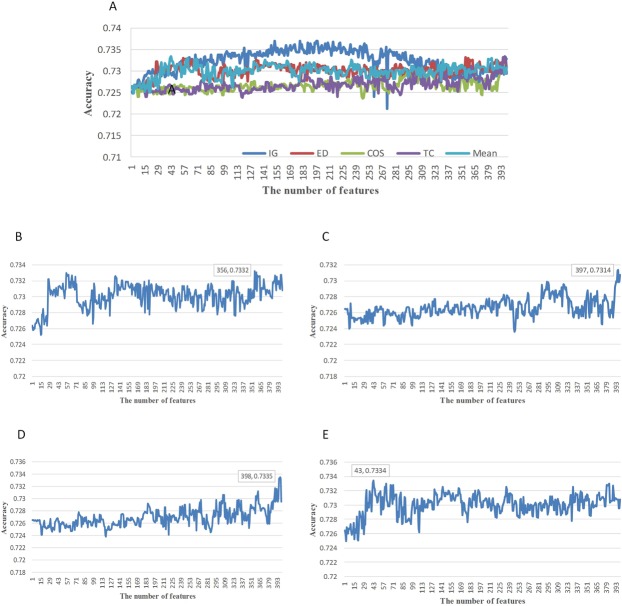
The predictive performance of different models and the comparison of their. (**A**) Comparison on the predictive performance of different feature selection methods. (**B**) The predictive performance of different models based on incremental feature selection of features sorted by distance ED of MRMD. (**C**) The predictive performance of different models based on incremental feature selection of features sorted by distance COS of MRMD. (**D**) The predictive performance of different models based on incremental feature selection of features sorted by distance TD of MRMD. (**E**) The predictive performance of different models based on incremental feature selection of features sorted by distance Mean of MRMD.

We used four distance methods ED, COS, TC and Mean of MRMD to find out the best feature vectors combination through using 10-fold cross-validation. The detailed predictive performances are listed in Fig. 4. When the distance function ED was adopted, its best Acc achieved 73.32% with 356 features. And when the distance functions are COS, TC and Mean, the predictive performance is the highest with 397, 398 and 43 features, respectively, whose corresponding predictive performance is 73.14%, 73.35% and 73.34%. Suppose that the total dimension of feature vector is 400, the influence of dimension reduction is not obvious when the distance function ED, COS and TC are used (the predictive performance is the best with 356, 397 and 398 features, respectively). However, the influence of dimension reduction is prominent when the Mean distance function is used (the predictive performance is the best with 43 features), which causes a lot of information lost in the feature vector. The best performances for different feature selection methods are listed in Supplementary Tables S8–11.

From Fig. 4A, we can see that although the performances of two methods, IG and four types of MRMD, are almost identical on the same datasets, Acc of IG has better advantageous. Meanwhile, its Acc is generally higher than that of MRMD method, including ED, COS, TC and Mean. Moreover, it has more advantages to achieve the dimensionality reduction of high-dimensional eigenvectors and unsure high Acc. From Fig. 4B–E, show the predictive performance of different dimensions eigenvectors are shown when MD is ED, COS, TC and Mean, respectively.

### Comparison with other methods

To make a fair and fast comparison, we compared the prediction performance of our predictor with GPS-SNO^2^, iSNO-PseAAC^20^, iSNO-ANBPB^21^, PSNO^22^, iSNO-AAPair^19^ on the Xu training dataset by running 10-fold cross-validation test 10 times. The results were shown in Table 3. Our constructed model exhibits the best prediction performance with Acc of 83.11%, which was 1.41% higher than the previous best-performing predictor iSNO-AAPair, and 7.44% higher than Acc achieved by PSNO. Our predictor also gave a MCC of 0.6617, which was 0.0317 higher than the method of iSNO-AAPair, and 0.1498 higher than PSNO. Furthermore, Sn of our predictor was 83.33%, which was 3.73% higher than Sn of iSNO-AAPair, and 9.18% higher than PSNO. This comparison indicates that the proposed model is indeed promising and could at least play a role that complements the existing state-of-the art methods in this field. In addition, we tested the predictive power of our model with the powers of the SNOSite^32^, iSNO-AAPair^19^, iSNO-PseAAC^20^, iSNO-ANBPB^21^ on the Li test dataset; and we also compared our model with the GPS-SNO^2^, iSNO-PseAAC^20^, iSNO-AAPair^19^, and PSNO^22^ methods on Xu test dataset. The performances of the above-mentioned models against two test datasets are summarized in Supplementary Tables S3 and S4. On the Li independent test dataset, our model captured proteins O00429 (site 367), P13221 (site 83), P43235 (site 139) as S-nitrosylation sites, while methods iSNO-AAPair and iSNO-PseAAC incorrectly predicted them as non-S-nitrosylation sites. On the Xu independent test dataset, our model captured proteins O70572 (site 176), P51174 (site 342), Q8VDG5 (site 308), Q9WVQ5 (site 146), P55060 (site 344) as S-nitrosylation sites, while models iSNO-PseAAC and GPS-SNO incorrectly predicted S-nitrosylation sites as non- S-nitrosylation sites. To show the prediction results clearly, we summarized Sn, Sp, ACC and MCC that was achieved by each model in Table 4. As it can be seen that our predictor achieved the performance with Sn of 60.47%, Sp of 77.69% and Acc of 73.17% on the Li test dataset. Among the other five methods, the best prediction performance was achieved by the method of Li *et al*., with Sn of 51.16%, Sp of 69.42% and Acc of 64.63%. Our method is obviously superior to other methods. However on Xu test dataset, our predictor achieved the prediction performance with the Sn of 64.20%, the Sp of 75.00%, and the Acc of 70.17%, which is only better than iSNO-PseAAC with Sn of 50.2%, Sp of 75.1% and Acc of 62.8%. The results show that our predictor outperformed previous methods in terms of precision. But on the Xu test set, the results are not ideal, which may be caused as a result of not considering the physical chemistry properties. In the future work, we will consider more compressive features and further optimize the feature combination approaches.

**Table 3:** Compare with other methods performance on the training dataset.

Dataset	Test Method	Methods	Sn (%)	Sp (%)	Acc (%)	MCC
Xu training dataset	the 10_fold cross-validation test	GPS-SNO	45.01	73.33	59.9	0.1915
		iSNO-PseAAC	67.01	68.15	67.62	0.3515
		iSNO-ANBPB	67.33	73.78	70.77	0.4146
		PSNO	74.15	77.04	75.67	0.5119
		iSNO-AAPair	79.60	84.10	81.70	0.6300
		Our predictor (maximum)	83.33	82.92	83.11	0.6617
		Our predictor (average)	72.16	74.90	73.54	0.4704
	jackknife test	IG-SCORE	73.60	75.93	74.82	0.4952

**Table 4:** Compare with other methods performance on the test datasets.

Dataset	Methods	Sn (%)	Sp (%)	Acc (%)	MCC
Li test dataset	SNOSite	74.42	28.1	40.24	0.0248
	iSNO-AAPair	27.91	80.17	66.46	0.0858
	Li *et al*.	51.16	69.42	64.63	0.1886
	iSNO-PseAAC	58.14	63.64	62.2	0.1940
	iSNO-ANBPB	74.12	59.5	63.41	0.2984
	Our predictor	60.47	77.69	73.17	0.3588
Xu test dataset	GPS-SNO	44.5	81.0	64.7	0.2800
	iSNO-PseAAC	50.2	75.2	62.8	0.3000
	iSNO-AAPair	79.6	84.1	81.7	0.6300
	PSNO	87.7	85.0	86.2	0.7200
	Our predictor	64.20	75.00	70.17	0.3942

## Conclusion

The prediction of SNO sites is essential for better understanding of the basic biological theory, clinical diagnosis as well as the pharmaceuticals. In this study, we introduce the IG which is a tool for the analysis of the importance of amino acid and its position used in feature extraction. Here, we focus on the characteristics of the amino acids with its sequence. Four out of the nine characteristics of the combination are related to the amino acid residues. The four types of features were screened using the IG method, and the best dimension of the feature vector was selected. Among these features, 180 important features were screened from the feature kmer-2 whose dimension was reduced from 400 to 180, with the best prediction performance, and Sn and Acc are reached 83.33% and 83.11%, respectively. Theoretically, there is a lot of information in the protein structure when compared with the simple sequences, and this will be considered in the future scope of the work. With the development of internet and big data era coming, constructing databases^33–40^ and establishing powerful webserver are the direction of bioinformatics. Thus, making it convenient to most experimental scientists

## Material and Methods

### Datasets

The datasets were constructed using those of Li *et al*.^22^ and Xu *et al*.^19,20^ (henceforth named the Li dataset and Xu dataset, respectively). As described previously^19,20,24^, these datasets were derived on the basis of the experimental verification of the protein S-nitrosylation sites. Xu training dataset consisted of 731 positive SNO sites as positive samples and 810 non-SNO cysteine sites as negative samples from the 438 proteins with <=40% sequence similarity. These samples were used for training our prediction model. The Xu test dataset consisted of 81 SNO sites and 100 non-SNO sites, and the Li test dataset included 43 SNO sites and 121 non-SNO sites. In this study, Xu and Li test datasets were applied to test the prediction performance of our model.

**Figure 5:**
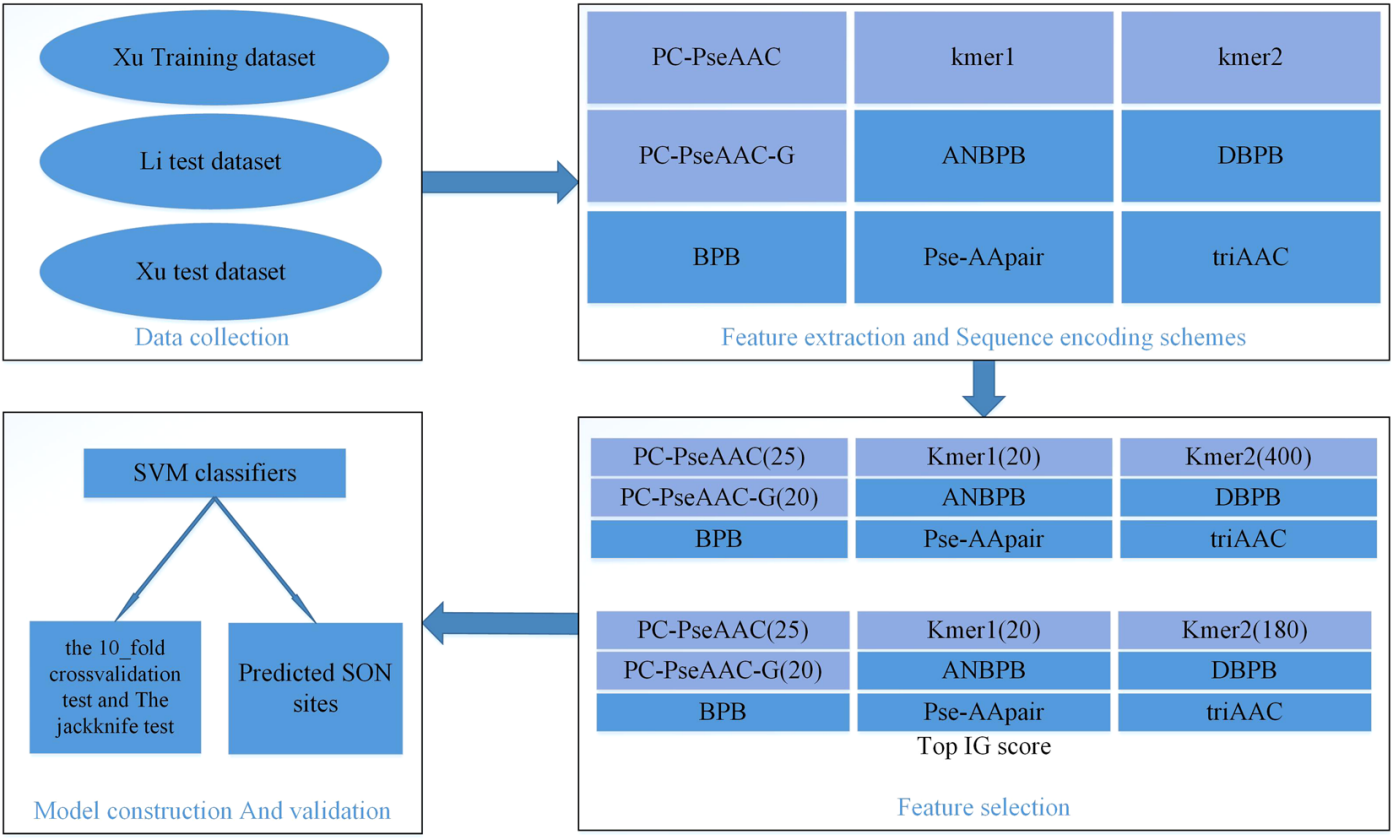
Flowchart of our predictor methodology.

Considering that we have a protein peptide sample P in our datasets, which can be generally formulated by:

1 $${\rm{P}}={R}_{-t}{R}_{-(t-1)}\ldots {R}_{-2}{R}_{-1}(C){R}_{+1}{R}_{+2}\ldots {R}_{+(t-1)}{R}_{+t}$$

where the subscript *t* is an integer, *R*_−*t*_ is the t-th downstream amino acid residue from cysteine(C), *R*_*t*_ the t-th upstream amino acid residue, and so forth. The peptide was termed as SNO or non-SNO peptide depending on whether its center is a SNO or non-SNO sites, respectively. P belonged to one of two categories *viz*. the SNO sites (positive data) or non-SNO sites (negative data). In the current study, we selected t = 10. If the upstream or downstream in a protein was less than 10, the lacking residues were filled using the dummy code X. Thus, the training dataset S was formulated as ($\cup$: in the set theory to formulate the union of datasets):

2 $${\rm{S}}={S}^{+}\cup {S}^{-}$$

where the positive dataset *S*^+^consisted of 731 SNO cysteine sites, while the negative dataset *S*^−^ contained 810 non-SNO cysteine sites; The test dataset T_*Li*_ and T_*Xu*_ was formulated as:

3 $${{\rm{T}}}_{Li}={{\rm{T}}}_{Li}^{+}\cup {{\rm{T}}}_{Li}^{-}$$

4 $${{\rm{T}}}_{Xu}={{\rm{T}}}_{Xu}^{+}\cup {{\rm{T}}}_{Xu}^{-}$$

where the positive dataset ${{\rm{T}}}_{Li}^{+}$ and ${{\rm{T}}}_{Xu}^{-}$ contained 43 and 81 SNO peptide fragments, respectively; while the negative dataset ${{\rm{T}}}_{Li}^{-}$ and ${{\rm{T}}}_{Xu}^{-}$ contained 121 and 100 non-SNO peptide fragments, respectively. For the reader’s convenience, the three datasets used in this study are given in Supplementary Tables S5–7. The schematic flowchart of our work is being shown in Fig. 5.

### Features extraction

#### Parallel correlation pseudo amino acid composition (PC-PseAAC)

PC-PseAAC^41^ is the feature extraction approach that incorporates the contiguous local and the global sequence-order information to obtain the feature vector for the protein sequence. Given a protein peptide P (Eq. 1), the PC-PseAAC feature vector for P is given by:

5 $${\rm{V}}={[{x}_{1},{x}_{2},\ldots ,{x}_{20},{x}_{21},\ldots ,{x}_{20+\lambda }]}^{T}$$

where

6 $${{\rm{x}}}_{u}=\{\begin{array}{l}\frac{{f}_{u}}{{\sum }_{i=1}^{20}\,{f}_{i}+w\,{\sum }_{j=1}^{\lambda }\,{{\Theta }}_{j}}(1\le u\le 20)\\ \frac{w{\Theta }_{u-20}}{{\sum }_{i=1}^{20}\,{f}_{i}+w\,{\sum }_{j=1}^{\lambda }\,{{\Theta }}_{j}}(20+1\le u\le 20+\lambda )\end{array}$$

where *f*_*i*_ (i = 1,2, …, 20) is the normalized occurrence frequency of the 20 amino acids in the protein P; the parameter λ is an integer, representing the highest counted rank (or tier) of the correlation along a protein sequence; w is the weight factor ranging from 0 to 1; and *Θ*_*j*_ (j = 1,2, …, 20) is the j-tier correlation factor reflecting the sequence-order correlation between all the j-th most contiguous residues along a protein chain, which is defined as:

7 $${{\Theta }}_{\lambda }=\frac{1}{L-\lambda }\,\sum _{i=1}^{L-\lambda }\,{\Theta }({R}_{i},{R}_{i+\lambda })\,(0 < \lambda  < L)$$

where the correlation function is given by:

8 $$\begin{array}{rcl}{\Theta }({R}_{i},{R}_{j}) & = & \frac{1}{3}\{{[{H}_{1}({R}_{j})-{H}_{1}({R}_{i})]}^{2}+{[{H}_{2}({R}_{j})-{H}_{2}({R}_{i})]}^{2}\\  &  & +\,{[M({R}_{j})-M({R}_{i})]}^{2}\}\end{array}$$

where ${H}_{1}({R}_{i})$, ${H}_{2}({R}_{i})$ and $M({R}_{i})\,$ are the hydrophobicity value, hydrophilicity value, and side-chain mass, respectively, of the amino acid *R*_*i*_. It should be noted that before substituting the values of hydrophobicity, hydrophilicity, and side-chain mass into Eq. 7, they are all subjected to a standard conversion as described by the following equation:

9 $${H}_{1}(i)=\frac{{H}_{1}^{0}(i)\,-\,{\sum }_{i=1}^{20}\,\frac{{H}_{1}^{0}(i)}{20}}{\sqrt{\frac{{\sum }_{i=1}^{20}\,{[{H}_{1}^{0}(i)-{\sum }_{i=1}^{20}\frac{{H}_{1}^{0}(i)}{20}]}^{2}}{20}}}$$

10 $${H}_{2}(i)=\frac{{H}_{2}^{0}(i)\,-\,{\sum }_{i=1}^{20}\,\frac{{H}_{2}^{0}(i)}{20}}{\sqrt{\frac{{\sum }_{i=1}^{20}\,{[{H}_{2}^{0}(i)-{\sum }_{i=1}^{20}\frac{{H}_{2}^{0}(i)}{20}]}^{2}}{20}}}$$

11 $$M(i)=\frac{{M}^{0}(i)\,-\,{\sum }_{i=1}^{20}\,\frac{{M}^{0}(i)}{20}}{\sqrt{\frac{{\sum }_{i=1}^{20}\,{[{M}^{0}(i)-{\sum }_{i=1}^{20}\frac{{M}^{0}(i)}{20}]}^{2}}{20}}}$$

where ${H}_{1}^{0}(i)$ and ${H}_{2}^{0}(i)$ represent the original hydrophobicity value and the original hydrophilicity value of the i-th amino acid respectively; and ${M}^{0}(i)$ is the mass of the i-th amino acid side chain.

#### General parallel correlation pseudo amino acid composition (PC-PseAAC_G)

The PC-PseAAC_G approach^42^, not only incorporates the comprehensive built-in indices extracted from the AAindex^43^, but also allows the users to upload their own indices to generate the PC-PseAAC_G feature vector. For a given a protein peptide P (Eq. 1), the PC-PseAAC_G feature vector of P is defined as:

12 $${\rm{V}}={[{x}_{1},{x}_{2},\ldots ,{x}_{20},{x}_{21},\ldots ,{x}_{20+\lambda }]}^{T}$$

where

13 $${{\rm{x}}}_{u}=\{\begin{array}{l}\frac{{f}_{u}}{{\sum }_{i=1}^{20}\,{f}_{i}+w\,{\sum }_{j=1}^{\lambda }\,{{\Theta }}_{j}}(1\le u\le 20)\\ \frac{w{{\Theta }}_{u-20}}{{\sum }_{i=1}^{20}\,{f}_{i}+w\,{\sum }_{j=1}^{\lambda }\,{{\Theta }}_{j}}(20+1\le u\le 20+\lambda )\end{array}$$

where *f*_*i*_ (i = 1,2, …, 20) is the normalized occurrence frequency of the 20 amino acids in the protein P; the parameter λ is an integer, representing the highest counted rank (or tier) of the correlation along a protein sequence; w is the weight factor ranging from 0 to 1; and *Θ*_*j*_ (j = 1,2,…,20) is called the j-tier correlation factor reflecting the sequence-order correlation between all the j-th most contiguous residues along a protein chain, which is defined as:

14 $${{\Theta }}_{\lambda }=\frac{1}{L-\lambda }\,\sum _{i=1}^{L-\lambda }\,{\Theta }({R}_{i},{R}_{i+\lambda })\,(0 < \lambda  < L)$$

In this case, the correlation function is given by:

15 $${\Theta }({R}_{i},{R}_{j})=\frac{1}{\mu }\,\sum _{u=1}^{\mu }\,{[{H}_{u}({R}_{i})-{H}_{u}({R}_{j})]}^{2}$$

where µ is the number of physicochemical indices considered; ${H}_{u}({R}_{i})$ is the u-th physicochemical index value of the amino acid *R*_*i*_; ${H}_{u}({R}_{j})$ is the u-th physicochemical index value for the amino acid *R*_*j*_. It should be noted that before substituting the physicochemical indices values into Eq. 14, they were also all subjected to a standard conversion as described by the following equation:

16 $${H}_{u}(i)=\frac{{H}_{u}^{0}(i)\,-\,{\sum }_{i=1}^{20}\,\frac{{H}_{u}^{0}(i)}{20}}{\sqrt{\frac{{\sum }_{i=1}^{20}\,{[{H}_{u}^{0}(i)-{\sum }_{i=1}^{20}\frac{{H}_{u}^{0}(i)}{20}]}^{2}}{20}}}$$

where ${H}_{u}^{0}(i)$ is the u-th original physicochemical value of the i-th amino acid.

#### Basic kmer (kmer)

Basic kmer^44^ is the simplest approach to represent the proteins by a numerical vector, in which the protein sequences are represented as the occurrence frequencies of k neighboring amino acids^45^. Given a protein sequence P (Eq. 1), the kmer feature vector of P is formulated as follows:

17 $${\rm{V}}({\rm{kmer}}\,-\,1)={[{x}_{1},{x}_{2},\ldots ,{x}_{i},\ldots ,{x}_{20}]}^{T}(0 < i\le 20)$$

18 $${\rm{V}}({\rm{kmer}}\,-\,2)={[{y}_{1},{y}_{2},\ldots ,{y}_{i},\ldots ,{y}_{400}]}^{T}(0 < i\le 400)$$

where *x*_*i*_ and *y*_*i*_ are the normalized occurrence frequency of the 20 amino acid residues and 400 dipeptides in the protein P, respectively.

#### Bi-Profile Bayes (BPB)

BPB^26^ comprehensively considers the information contained in the two aspects of positive and negative samples that have been successfully applied in many fields of bioinformatics and has made effective predictions^26,46–48^. Given a protein peptide P (Eq. 1), the BPB feature vector of P is defined as:

19 $${\rm{V}}={[{x}_{1},{x}_{2},\ldots ,{x}_{n},{x}_{n+1},\ldots ,{x}_{2n}]}^{T}$$

where V is the posterior probability vector; ${x}_{1},{x}_{2},\ldots ,{x}_{n}$ represents the posterior probability of each amino acid at each position in positive peptide sequence datasets; ${x}_{n+1},\ldots ,{x}_{2n}$ represents the posterior probability of each amino acid at each position in negative peptide sequence datasets. Two position-specific profiles for final model training, positive position-specific profiles and negative position-specific profiles, were generated by calculating the frequency of each amino acid at each position in the positive datasets and negative datasets, respectively.

#### Double Bi-Profile Bayes (DBPB)

DBPB is an improvement of BPB that was proposed by Shao *et al*.^24^. As mentioned above, BPB is the posterior probability of each single amino acid at each position in the positive and negative datasets, while DBPB is the posterior probability of each two adjacent amino acids at each position in the positive and negative datasets. Given a protein sequence P (Eq. 1), the DBPB feature vector of P is defined as:

20 $${\rm{V}}={[{x}_{1},{x}_{2},\ldots ,{x}_{n-1},{x}_{(n-1)+1},\ldots ,{x}_{2(n-1)}]}^{T}$$

where V is the posterior probability vector; ${x}_{1},{x}_{2},\ldots ,{x}_{n-1}$ that represents the posterior probability of each dipeptide at each position in positive peptide sequence datasets; ${x}_{(n-1)+1},\ldots ,{x}_{2(n-1)}$ represents the posterior probability of each dipeptide at each position in the negative peptide sequence datasets. Two position-specific profiles for the final model training, positive position-specific profile and negative position-specific profile were generated by calculating the frequency of each amino acid pair at each position in the positive datasets and negative datasets, respectively.

#### Adapted Normal distribution Bi-Profile Bayes (ANBPB)

ANBPB^21,49^ is the improvement of BPB in another aspect. Given a protein sequence P (Eq. 1), the ANBPB feature vector of P is defined as:

21 $${\rm{V}}={[{p}_{1},{p}_{2},\ldots ,{p}_{n},{p}_{n+1},\ldots ,{p}_{2n}]}^{T}$$

where ${p}_{1},{p}_{2},\ldots ,{p}_{n}$ is the posterior probability of each amino acid at each position in positive peptide sequences datasets; ${p}_{n+1},\ldots ,{p}_{2n}$ is defined based on the posterior probability of each amino acid at each position in negative peptide sequences datasets. The posterior probability ${p}_{1},{p}_{2},\ldots ,{p}_{2n}$ was coded by the adapted normal distribution as follows:

22 $${p}_{i}={\rm{\phi }}(x)=\frac{1}{\sqrt{2\pi }}\,{\int }_{-\infty }^{x}\,{e}^{-\frac{{t}^{2}}{2}}{\rm{dt}}$$

where φ(*x*) is the standard normal distribution function and the detailed description of the formula is given^21,49^.

#### Incorporating Amino Acid Pairwise (IAAPair)

The posterior probability of every two adjacent amino acids and each two next nearest amino acids at each position in the positive peptide sequence datasets is subtracted from in the negative peptide sequence datasets^19^. Given a protein sequence P (Eq. 1), the IAAPair feature vector of P is defined as:

23 $${\rm{V}}={[{p}_{1},{p}_{2},\ldots ,{p}_{j},\ldots ,{p}_{[(n-1)+(n-2)]}]}^{T}$$

24 $$+\,{\rm{V}}={[\begin{array}{ccc}+{{p}^{0}}_{1,1},+{{p}^{0}}_{1,2}, & \cdots  & ,+{{p}^{0}}_{1,n-1},\\ \vdots  & \ddots  & \vdots \\ +{{p}^{0}}_{{21}^{2},1},+{{p}^{0}}_{{21}^{2},2}, & \cdots  & ,+{{p}^{0}}_{{21}^{2},n-1},\end{array}\begin{array}{ccc}+{{p}^{1}}_{1,1},+{{p}^{1}}_{1,2}, & \cdots  & ,+{{p}^{1}}_{1,n-2},\\ \vdots  & \ddots  & \vdots \\ +{{p}^{1}}_{{21}^{2},1},+{{p}^{1}}_{{21}^{2},2}, & \cdots  & ,+{{p}^{1}}_{{21}^{2},n-2},\end{array}]}^{T}$$

25 $$-\,{\rm{V}}={[\begin{array}{ccc}-{{p}^{0}}_{1,1},-{{p}^{0}}_{1,2}, & \cdots  & ,-{{p}^{0}}_{1,n-1},\\ \vdots  & \ddots  & \vdots \\ -{{p}^{0}}_{{21}^{2},1},-{{p}^{0}}_{{21}^{2},2}, & \cdots  & ,-{{p}^{0}}_{{21}^{2},n-1},\end{array}\begin{array}{ccc}-{{p}^{1}}_{1,1},-{{p}^{1}}_{1,2}, & \cdots  & ,-{{p}^{1}}_{1,n-2},\\ \vdots  & \ddots  & \vdots \\ -{{p}^{1}}_{{21}^{2},1},-{{p}^{1}}_{{21}^{2},2}, & \cdots  & ,-{{p}^{1}}_{{21}^{2},n-2},\end{array}]}^{T}$$

26 $${\rm{V}}=(\,+\,{\rm{V}})-(\,-\,{\rm{V}})$$

27 $${p}_{j}=\{\begin{array}{ll}\pm {{p}^{0}}_{1,j} & when\,{R}_{t}{R}_{t+1}=AA\,and\,1\le j\le n-1\\ \pm {{p}^{0}}_{2,j} & when\,{R}_{t}{R}_{t+1}=AC\,and\,1\le j\le n-1\\  & \vdots \,\,\,\,\,\,\,\vdots \\ \pm {{p}^{0}}_{({21}^{2}-1),j} & when\,{R}_{t}{R}_{t+1}=XY\,and\,1\le j\le n-1\\ \pm {{p}^{0}}_{{21}^{2},j} & when\,{R}_{t}{R}_{t+1}=XX\,and\,1\le j\le n-1\\ \pm {{p}^{1}}_{1,j} & when\,{R}_{t}{R}_{t+1}=AA\,and\,n\le j\le [(n-1)+(n-2)]\\ \pm {{p}^{1}}_{2,j} & when\,{R}_{t}{R}_{t+1}=AC\,and\,n\le j\le [(n-1)+(n-2)]\\  & \,\,\vdots \,\,\,\,\,\,\,\vdots \\ \pm {{p}^{1}}_{({21}^{2}-1),j} & when\,{R}_{t}{R}_{t+1}=XY\,and\,n\le j\le [(n-1)+(n-2)]\\ \pm {{p}^{1}}_{{21}^{2},j} & when\,{R}_{t}{R}_{t+1}=XX\,and\,n\le j\le [(n-1)+(n-2)]\end{array}$$

where V is the posterior probability vector (in this feature, the C in the middle of peptide sequence must not be omitted). When $1\le j\le n\,-\,1$ *p*_*j*_ is the representative posterior probability of every two nearest amino acids, and when $n\le j\le [(n-1)+(n-2)]$ *p*_*j*_ is the representative posterior probability of each two next nearest amino acids. +*p*_*i*,*j*_ and −*p*_*i*,*j*_ represent the posterior probability of every two nearest amino acids and each two next nearest amino acids at each position in positive and negative peptide sequence datasets, respectively. $\pm {p}_{i,j}=(\,+\,{p}_{i,j})-(\,-\,{p}_{i,j})$ is the feature vector.

#### Position-specific Tri-Amino Acid Propensity (PSTAAP)

The posterior probability of every three adjacent amino acids at each position in the positive peptide sequence datasets is subtracted from in the negative peptide sequence datasets^20,27^. Given a cysteine peptide fragment P (Eq. 1), the feature vector of PSTAAP for P is defined as follows:

28 $${\rm{V}}={[{p}_{1},{p}_{2},\ldots ,{p}_{j},\ldots ,{p}_{n-2}]}^{T}$$

29 $$+{\rm{V}}={[\begin{array}{ccc}+{p}_{1,1},+{p}_{1,2}, & \cdots  & ,+{p}_{1,n-2}\\ \vdots  & \ddots  & \vdots \\ +{p}_{{21}^{3},1},+{p}_{{21}^{3},2}, & \cdots  & +{p}_{{21}^{3},n-2}\end{array}]}^{T}$$

30 $$-{\rm{V}}={[\begin{array}{ccc}-{p}_{1,1},-{p}_{1,2}, & \cdots  & ,-{p}_{1,n-2}\\ \vdots  & \ddots  & \vdots \\ -{p}_{{21}^{3},1},-{p}_{{21}^{3},2}, & \cdots  & ,-{p}_{{21}^{3},n-2}\end{array}]}^{{T}^{T}}$$

31 $${\rm{V}}=(\,+\,{\rm{V}})-(\,-\,{\rm{V}})$$

32 $${p}_{j}=\{\begin{array}{ll}\pm {p}_{1,j} & when\,{R}_{t}=AAA\\ \pm {p}_{2,j} & when\,{R}_{t}=AAC\\ \vdots  & \,\,\,\,\,\,\vdots \\ \pm {p}_{({21}^{3}-1),j} & when\,{R}_{t}=XXY\\ \pm {p}_{{21}^{3},j} & when\,{R}_{t}=XXX\end{array}$$

where V is the posterior probability vector; +*p*_*i*,*j*_ represents the posterior probability of each tri-amino acids at each position in the positive dataset; −*p*_*i*,*j*_ represents the posterior probability of each tri-amino acids at each position in the negative dataset; $\pm {p}_{j}=(\,+\,{p}_{i,j})-(\,-\,{p}_{i,j})$ is the feature vector.

It should be indicated that recently a very powerful web-server called ‘Pse-in-One’^25^, and its updated version ‘Pse-in-One2.0’^45^ have been established and can be used to generate any desired feature vectors for protein/peptide and DNA/RNA sequences according to the user study needs or desires. In the current study, the feature vectors PC-PseAAC, PC-PseAAC_General, and basic kmer are obtained from the web-server.

### Information Gain (IG)

The IG^44,46–48^ method is usually used to rank the importance of positions and amino acid residues. IG measures the decrease in entropy when a given feature is used to group values of another (class) feature. The entropy of a feature X is defined by:

where {*x*_*i*_} is a set of values of X and *P*(*x*_*i*_) is the prior probability of *x*_*i*_. If Y is considered as another feature, the conditional entropy of X is defined as:

33 $${\rm{H}}({\rm{X}}|{\rm{Y}})=-\,\sum _{j}\,P({y}_{j})\,\sum _{i}\,P({x}_{i}|{y}_{j})\,{lo}{{g}}_{2}\,(({x}_{i}|{y}_{j}))$$

where $P({x}_{i}|{y}_{j})$ is the posterior probability of X with the value *y*_*j*_ of Y. The amount by which the entropy of X decreases reflects the additional information about X provided by Y and is called the information gain:

34 $${\rm{IG}}({\rm{X}}|{\rm{Y}})={\rm{H}}({\rm{X}})\,-\,{\rm{H}}({\rm{X}}|{\rm{Y}})$$

According to this measure, Y has a stronger correlation with X than with Z, if IG (X|Y) > IG (Z |Y). It is obvious that Y represents the amino acid type, when extracting the IG score for positions. On the other hand, Y represents the amino acid frequency, when extracting IG score for amino acids.

Calculating IG score of positions and amino acid residues:

1. The importance of positions: The 20 amino acid residues (A, C, D, E, F, G, H, I, K, L, M, N, P, Q, R, S, T, V, W, and Y) were coded into digits from 1 to 20. The query sequences segments were coded into an X-dimension digital sequence (protein).
2. The importance of amino acid residues: The amino acid frequency in the surrounding sequence query site (the site itself is not counted) was calculated. The query sequences were also coded into a 20-dimension feature.
3. Calculation of the IG score for positions by (1) and the IG procedure was performed. Subsequently, the calculation of the IG score for amino acid residues by (2) and the IG procedure was done. Then, we ranked the corresponding positions and amino acid residues by their IG score and selected the key positions and key amino acid residues.

In this work, we used the IG score to calculate the importance of amino acid residues:

35 $$\begin{array}{rcl}{\rm{IG}}({{\rm{X}}|{\rm{Y}}}_{i}) & = & {\rm{H}}({\rm{X}})-{\rm{H}}({{\rm{X}}|{\rm{Y}}}_{i})\\  & = & -\,\sum _{x\in (0,1)}\,P(x)\,lo{g}_{2}((x))\,\sum _{{y}_{j}\in (0,1)}\,P({y}_{i})\\  &  & \times \,\sum _{x\in (0,1)}\,P(x|{y}_{j})\,lo{g}_{2}(({x}_{i}|{y}_{j}))\end{array}$$

Equation (36) is divided into two parts, the former is the entropy H(X) of class X, and the latter part is the conditional entropy ${\rm{H}}({{\rm{X}}|{\rm{Y}}}_{i})$ of X a given amino acid Y.

Suppose the number of training samples is N. Initially, we count each training sample. Subsequently, if each characteristic *y*_*j*_ is added in the training sample x, two times will be counted once:

36 $$P({y}_{j}=1)=\frac{count({y}_{j}=1)}{N}$$

37 $$P({y}_{j}=0)=1-P({y}_{j}=1)$$

38 $$P(X=0|{y}_{j}=1)=\frac{count(X=0,{y}_{j}=1)}{count({y}_{j}=1)}$$

39 $$P(X=1|{y}_{j}=1)=\frac{count(X=1,{y}_{j}=1)}{count({y}_{j}=1)}$$

40 $$P(X=0|{y}_{j}=0)=\frac{count(X=0,{y}_{j}=0)}{count({y}_{j}=0)}$$

41 $$P(X=1|{y}_{j}=0)=\frac{count(X=1,{y}_{j}=0)}{count({y}_{j}=0)}.$$

### Max-Relevance-Max-Distance (MRMD)

MDMR^31^ is a feature selection method for reducing dimensionalities, which can be further divided into two aspects.

1. One is the relevance between sub-feature set and target class. Here, Pearson’s correlation coefficient is exploited to measure the relevance. With the increase of Pearson’s correlation coefficient, the relevant between feature and target class also increases.
2. The other is redundancy of sub-feature set. Three kinds of distance functions are utilized to calculate the redundancy. The larger the feature distance, the lower the redundancy for sub-feature set becomes.

The features with large sum of relevance and distance would be chosen as the ultimate sub-feature set. Finally, the sub-feature set generated by MRMD has low redundancy and strong relevance with the target class.

In order to describe the algorithm clearly, we listed some functions in following section. Given the input datasets tabled as N instances, M features ${\rm{F}}=\{{{\rm{f}}}_{i},i=1,\ldots ,M\}$ and the target class C, the aim is to find a subspace of *M* features, which is selected from the *M* dipeptides original space, and makes the greatest contribution to classify the target class C.

#### Max-relevance (MR)

Making the greatest contribution for classifying the target class condition and this often requires the maximal relevance for the target class C on the subspace, which needs us to select a feature set with the highest relevance to target class C. We use the Pearson’s correlation coefficient to measure positive correlation and negative correlation. Because it is suitable for calculating continuous variables and easy to implement, Pearson’s correlation coefficient is adopted as the measure of relevance between feature and target class C.

The value of MR for feature *i* can be defined as follows.

42 $${\rm{mac}}\,{{\rm{MR}}}_{i}=|{\rm{PCC}}(\mathop{\to }\limits_{{F}_{i}}\mathop{\to }\limits_{{C}_{i}})|\,(1\le i\le M)$$

where $\mathop{\to }\limits_{{F}_{i}}$ is a vector composed from *i*th features from each instance, and $\mathop{\to }\limits_{{C}_{i}}$ is also a vector whose every element comes from the target class C of each instance. Their Pearson’s correlation coefficient is defined as ${\rm{PCC}}(\mathop{\to }\limits_{{F}_{i}}\mathop{\to }\limits_{{C}_{i}})$.

#### Max-Distance (MD)

MDMR proposed a new approach to realize Max-Redundancy based on distance function, namely maximal distance, to measure the level of similarity between two feature vectors. There are three types of distance functions that can be chosen, which are Euclidean distance, cosine similarity and Tanimoto coefficient. Compared with the commonly used methods, Euclidean distance is easier to calculate. As compared to the Euclidean distance, cosine similarity focuses on the angle between two vectors. The last one, Tanimoto coefficient, is also called Jaccard coefficient in the broad sense. Under the binary condition, it is similar to Jaccard coefficient. For each feature, its value of distance defined as follows is based on t three types of distance functions mentioned above. According to the following formula, we can obtain their values for the feature *i* ($1\le i\le M$) ED_*i*_, COS_*i*_ and TC_*i*_, respectively.

43 $${{\rm{ED}}}_{i}=\frac{1}{M-1}\,\sum \,ED(\mathop{\to }\limits_{{F}_{i}}\mathop{\to }\limits_{{F}_{k}})\,(1\le k\le M,k\ne i)$$

44 $${\mathrm{COS}}_{i}=\frac{1}{M-1}\,\sum \,COS(\mathop{\to }\limits_{{F}_{i}}\mathop{\to }\limits_{{F}_{k}})\,(1\le k\le M,k\ne i)$$

45 $${{\rm{TD}}}_{i}=\frac{1}{M-1}\,\sum \,TD(\mathop{\to }\limits_{{F}_{i}}\mathop{\to }\limits_{{F}_{k}})\,(1\le k\le M,k\ne i)$$

From three formulas above, we have four ways to obtain the final value of MD.

46 $$max\,{{\rm{MD}}}_{i}={{\rm{ED}}}_{i}\,(1\le i\le M)$$

47 $$max\,{{\rm{MD}}}_{i}={\mathrm{COS}}_{i}\,(1\le i\le M)$$

48 $$max\,{{\rm{MD}}}_{i}={{\rm{TC}}}_{i}(1\le i\le M)$$

49 $$mean\,{{\rm{MD}}}_{i}=\frac{1}{3}({{\rm{ED}}}_{i}+{\mathrm{COS}}_{i}+{{\rm{TC}}}_{i})\,(1\le i\le M)$$

We can obtain top *m* features which are considered to be the sub-feature set with minimal redundancy by MD.

#### MRMD

The criterion used for combining the two constraints above is called “Max-Relevance-Max-Distance” (MRMD). After having done all the above preparations, we could start to select the features subspace. The algorithm optimizes the following condition.

For a specific problem, the condition for feature selection take into consideration that the MR is not as important as MD. Therefore, the variables w_*r*_ $(1\le {{\rm{w}}}_{r}\le M)$ and w_*d*_ $(1\le {{\rm{w}}}_{d}\le M)$ are the weights of MR and MD, respectively.

## Supplementary information

Supplementary Table

## References

[1] Foster MW, Hess DT, Stamler JS (2009). Protein S-nitrosylation in health and disease: a current perspective. Trends Mol Med.

[2] Xue Y (2010). GPS-SNO: Computational Prediction of Protein S-Nitrosylation Sites with a Modified GPS Algorithm. PloS one.

[3] Lim KH, Ancrile BB, Kashatus DF, Counter CM (2008). Tumour maintenance is mediated by eNOS. Nature.

[4] Li F (2007). Regulation of HIF-1 alpha stability through S-nitrosylation. Mol Cell.

[5] Burgoyne JR, Eaton P (2010). A Rapid Approach for the Detection, Quantification, And Discovery Of Novel Sulfenic Acid Or S-Nitrosothiol Modified Proteins Using a Biotin-Switch Method. Method Enzymol.

[6] Jaffrey SR, Erdjument-Bromage H, Ferris CD, Tempst P, Snyder SH (2001). Protein S-nitrosylation: a physiological signal for neuronal nitric oxide. Nat Cell Biol.

[7] Gross SS (2006). SNOSID, a proteomic method for identification of cysteine S-nitrosylation sites in complex protein mixtures. Nitric Oxide-Biol Ch.

[8] Greco TM (2006). Identification of S-nitrosylation motifs by site-specific mapping of the S-nitrosocysteine proteome in human vascular smooth muscle cells. P Natl Acad Sci USA.

[9] Derakhshan B, Wille PC, Gross SS (2007). Unbiased identification of cysteine S-nitrosylation sites on proteins. Nat Protoc.

[10] Forrester MT (2009). Proteomic analysis of S-nitrosylation and denitrosylation by resin-assisted capture. Nat Biotechnol.

[11] Hess DT, Matsumoto A, Nudelman R, Stamler JS (2001). S-nitrosylation: spectrum and specificity. Nat Cell Biol.

[12] Lindermayr C, Saalbach G, Durner J (2005). Proteomic identification of S-nitrosylated proteins in Arabidopsis thaliana. Comp Biochem Phys A.

[13] Kuncewicz T, Sheta EA, Goldknopf IL, Kone BC (2003). Proteomic analysis of S-nitrosylated proteins in mesangial cells. Mol Cell Proteomics.

[14] Huang B, Chen SC, Wang DL (2009). Shear flow increases S-nitrosylation of proteins in endothelial cells. Cardiovasc Res.

[15] Lefievre L (2007). Human spermatozoa contain multiple targets for protein S-nitrosylation: An alternative mechanism of the modulation of sperm function by nitric oxide?. Proteomics.

[16] Foster MW, Forrester MT, Stamler JS (2009). A protein microarray-based analysis of S-nitrosylation. P Natl Acad Sci USA.

[17] Chen W, Yang H, Feng P, Ding H, Lin H (2017). iDNA4mC: identifying DNA N4-methylcytosine sites based on nucleotide chemical properties. Bioinformatics.

[18] Li YX, Shao YH, Jing L, Deng NY (2011). An Efficient Support Vector Machine Approach for Identifying Protein S-Nitrosylation Sites. Protein Peptide Lett.

[19] Xu Y, Shao XJ, Wu LY, Deng NY, Chou KC (2013). iSNO-AAPair: incorporating amino acid pairwise coupling into PseAAC for predicting cysteine S-nitrosylation sites in proteins. Peerj.

[20] Xu Y, Ding J, Wu LY, Chou KC (2013). iSNO-PseAAC: Predict Cysteine S-Nitrosylation Sites in Proteins by Incorporating Position Specific Amino Acid Propensity into Pseudo Amino Acid Composition. PloS one.

[21] Jia CZ, Lin X, Wang ZP (2014). Prediction of Protein S-Nitrosylation Sites Based on Adapted Normal Distribution Bi-Profile Bayes and Chou’s Pseudo Amino Acid Composition. Int J Mol Sci.

[22] Zhang J, Zhao XW, Sun PP, Ma ZQ (2014). PSNO: Predicting Cysteine S-Nitrosylation Sites by Incorporating Various Sequence-Derived Features into the General Form of Chou’s PseAAC. Int J Mol Sci.

[23] Lin H (2012). The prediction of protein structural class using averaged chemical shifts. J Biomol Struct Dyn.

[24] Li BQ, Hu LL, Niu S, Cai YD, Chou KC (2012). Predict and analyze S-nitrosylation modification sites with the mRMR and IFS approaches. J Proteomics.

[25] Liu B (2015). Pse-in-One: a web server for generating various modes of pseudo components of DNA, RNA, and protein sequences. Nucleic Acids Res.

[26] Shao JL, Xu D, Tsai SN, Wang YF, Ngai SM (2009). Computational Identification of Protein Methylation Sites through Bi-Profile Bayes Feature Extraction. PloS one.

[27] Xu Y, Wen X, Shao XJ, Deng NY, Chou KC (2014). iHyd-PseAAC: Predicting Hydroxyproline and Hydroxylysine in Proteins by Incorporating Dipeptide Position-Specific Propensity into Pseudo Amino Acid Composition. Int J Mol Sci.

[28] Chen XX (2016). Identification of Bacterial Cell Wall Lyases via Pseudo Amino Acid Composition. BioMed research international.

[29] He W, Jia C, Duan Y, Zou Q (2018). 70ProPred: a predictor for discovering sigma70 promoters based on combining multiple features. BMC systems biology.

[30] Jia C, Yang Q, Zou Q (2018). NucPosPred: Predicting species-specific genomic nucleosome positioning via four different modes of general PseKNC. Journal of theoretical biology.

[31] Zou Q, Zeng JC, Cao LJ, Ji RR (2016). A novel features ranking metric with application to scalable visual and bioinformatics data classification. Neurocomputing.

[32] Lee TY, Chen YJ, Lu TC, Huang HD, Chen YJ (2011). SNOSite: Exploiting Maximal Dependence Decomposition to Identify Cysteine S-Nitrosylation with Substrate Site Specificity. PloS one.

[33] Cui TY (2018). MNDR v2.0: an updated resource of ncRNA-disease associations in mammals. Nucleic Acids Res.

[34] Jiang J, Xing F, Zeng XX, Zou Q (2018). RicyerDB: A Database For Collecting Rice Yield-related Genes with Biological Analysis. Int J Biol Sci.

[35] Liang ZY (2017). Pro54DB: a database for experimentally verified sigma-54 promoters. Bioinformatics.

[36] Yi Y (2017). RAID v2.0: an updated resource of RNA-associated interactions across organisms. Nucleic Acids Res.

[37] Zhang T (2017). RNALocate: a resource for RNA subcellular localizations. Nucleic Acids Res.

[38] Manavalan B, Shin TH, Lee G (2018). PVP-SVM: Sequence-Based Prediction of Phage Virion Proteins Using a Support Vector Machine. Front Microbiol.

[39] Cheng JH (2018). Prediction of bacteriophage proteins located in the host cell using hybrid features. Chemometr Intell Lab.

[40] He WY, Ju Y, Zeng XX, Liu XR, Zou Q (2018). Sc-ncDNAPred: A Sequence-Based Predictor for Identifying Non-coding DNA in Saccharomyces cerevisiae. Front Microbiol.

[41] Chou KC (2001). Prediction of protein cellular attributes using pseudo-amino acid composition. Proteins.

[42] Cao DS, Xu QS, Liang Y (2013). Z. propy: a tool to generate various modes of Chou’s PseAAC. Bioinformatics.

[43] Kawashima S, Kanehisa M (2000). AAindex: Amino acid index database. Nucleic Acids Res.

[44] Liu B, Wang XL, Lin L, Dong QW, Wang X (2008). A discriminative method for protein remote homology detection and fold recognition combining Top-n-grams and latent semantic analysis. Bmc Bioinformatics.

[45] Liu B, Wu H, Chou K-C (2017). Pse-in-One 2.0: An Improved Package of Web Servers for Generating Various Modes of Pseudo Components of DNA, RNA, and Protein Sequences. Natural Science.

[46] Zhao, X. W. *et al*. Identification of Protein Pupylation Sites Using Bi-Profile Bayes Feature Extraction and Ensemble Learning. *Math Probl Eng*, Artn 283129, 10.1155/2013/283129 (2013).

[47] Jia CZ, Liu TA, Chang AK, Zhai YY (2011). Prediction of mitochondrial proteins of malaria parasite using bi-profile Bayes feature extraction. Biochimie.

[48] Jia CZ, He WY, Yao YH (2017). OH-PRED: prediction of protein hydroxylation sites by incorporating adapted normal distribution bi-profile Bayes feature extraction and physicochemical properties of amino acids. J Biomol Struct Dyn.

[49] Jia CZ, Liu T, Wang ZP (2013). O-GlcNAcPRED: a sensitive predictor to capture protein O-GlcNAcylation sites. Mol Biosyst.

